# P-1376. Impact of COVID-19 and Mpox on Inpatient Screening of STIs

**DOI:** 10.1093/ofid/ofae631.1552

**Published:** 2025-01-29

**Authors:** Samara Levine, Brian Carpenter, Amber C Streifel, Alexandria Shonk, Cara D Varley

**Affiliations:** OHSU, Portland, Oregon; Oregon Health and Science University, Portland, Oregon; Oregon Health and Science University, Portland, Oregon; OHSU-PSU School of Public Health, Portland, Oregon; Oregon Health & Science University, Portland, OR

## Abstract

**Background:**

Sexually transmitted infections (STIs) continue to pose a considerable public health challenge, with a notable uptick in STI rates over the last few years. Access to care, both inpatient and outpatient, was disrupted during COVID-19 with a subsequent increase in STIs reported in many areas, followed by additional strain on services with the emergence of Mpox in Oregon.

**Figure:**
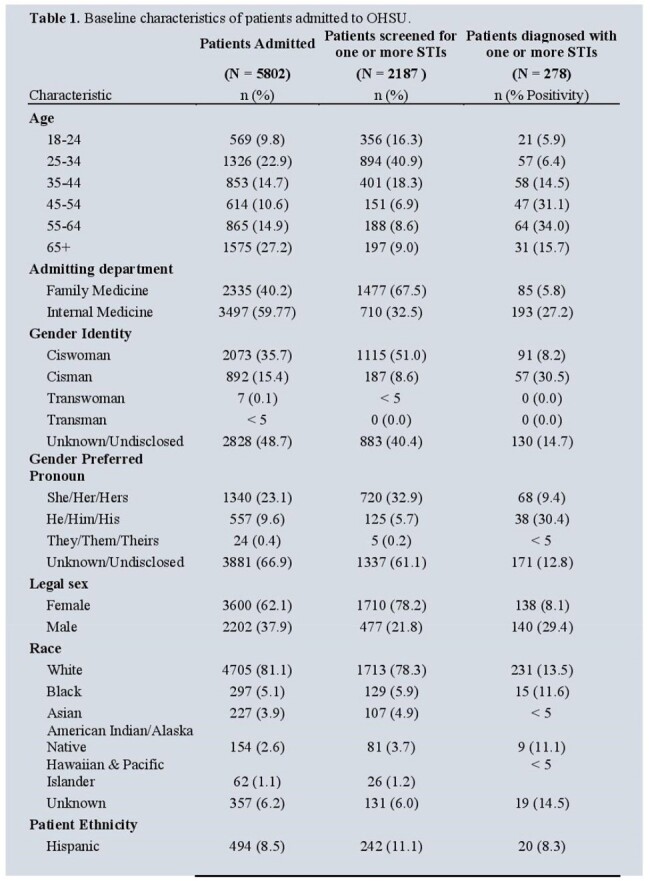

Abbreviations: sexually transmitted infection (STI)

STIs include chlamydia, gonorrhea, syphilis, human immunodeficiency virus (HIV), and hepatitis C

**Methods:**

We conducted a retrospective cohort of hospitalized patients at the Oregon Health & Science University (OHSU) Internal Medicine and Family Medicine services to evaluate STI screening and diagnoses (including chlamydia, gonorrhea, HIV, HCV and syphilis) across distinct periods: pre-COVID-19 pandemic (1/1/2018-2/29/2020), during the COVID-19 pandemic (3/1/2020-7/31/2022), and amidst the Mpox surge (8/1/2022-3/1/2023). We calculated the proportion screened, percent positivity by time and compared proportions between our pre-designated periods using the two-sample test of equal proportions.

**Figure:**
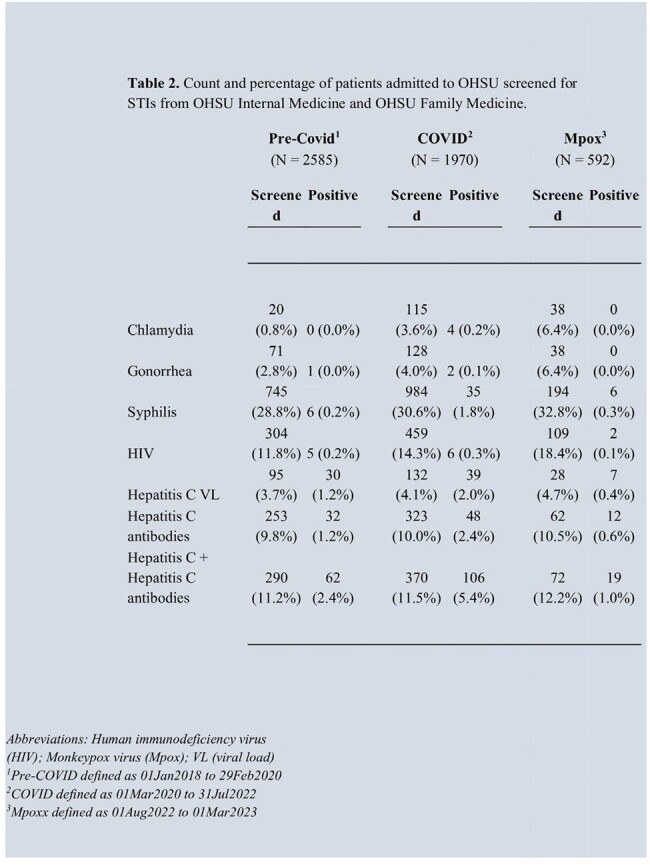

**Results:**

During the study period, 5802 patients were admitted to OHSU. Most were White (4705, 81.1%), non-Hispanic (5308, 91.5%) and Female sex (3600, 62.1%). In total, 2151 (37%) were screened for one or more STIs. HIV and syphilis were the most frequently screened STIs at 15.0% and 33.1%. The highest proportion of STI screenings were among those 25-44 years at 40.9% with the highest percent positivity in those 55-64 years at 34.0%. There was a statistically significant increase in proportion screened for all STIs (see Table 2), along with percent positivity for syphilis (P<0.01) between pre-COVID-19 and COVID-19 period, however this trend did not consistently continue after emergence of Mpox.

**Figure:**
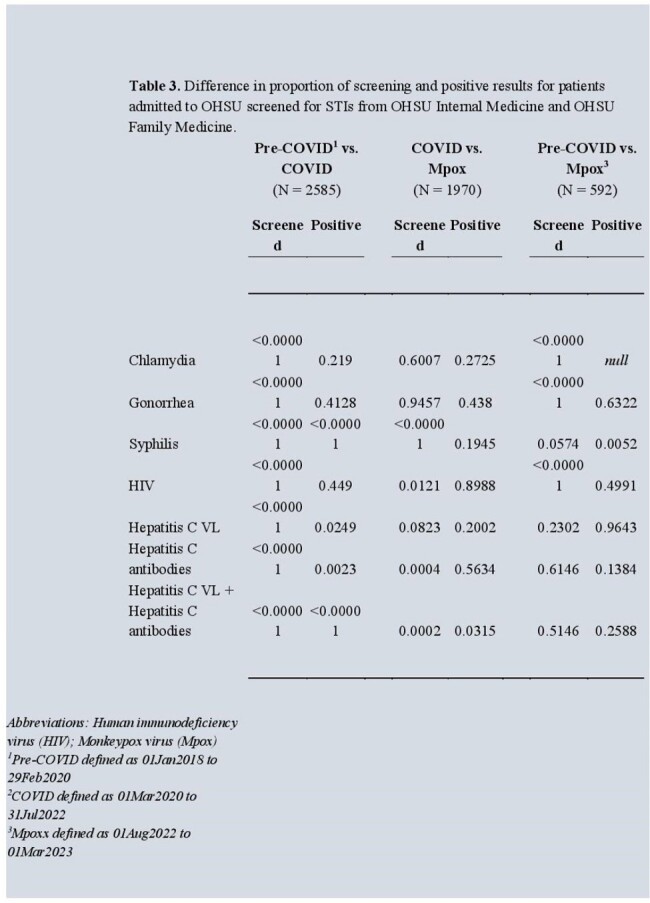

**Conclusion:**

The proportion of inpatient STI screening is low for patients admitted to internal medicine and family medicine services with percent positivity by age ranging from 5.9-34.0%. We observed an overall increase in proportion screened during the COVID-19 period.

**Disclosures:**

**All Authors**: No reported disclosures

